# Dividing CKD stage 3 into G3a and G3b could better predict the prognosis of IgA nephropathy

**DOI:** 10.1371/journal.pone.0175828

**Published:** 2017-04-17

**Authors:** Jun-Jun Zhang, Gui-Zhen Yu, Zhao-Hui Zheng, Ya-Fei Liu, Yang-Yang Du, Song-Xia Quan, Yu-Jie Liu, Ji-Cheng Lv, Hong Zhang

**Affiliations:** 1 Department of Nephrology, The First Affiliated Hospital of Zhengzhou University, Zhengzhou, Henan, PR China; 2 Research Institute of Nephropathy, Zhengzhou University, Zhengzhou, Henan, China; 3 Department of Rheumatism Immunity, The First Affiliated Hospital of Zhengzhou University, Zhengzhou, Henan, China; 4 Department of Nephrology and Rheumatology, Children’s Hospital of Zhengzhou City, Zhengzhou, Henan, China; 5 Renal Division, Peking University First Hospital, Beijing, China; University of Utah School of Medicine, UNITED STATES

## Abstract

Chronic kidney disease (CKD) stage 3 was divided into stage G3a and stage G3b in the 2013 Kidney Disease Improving Global Outcomes guidelines. Whether it is appropriate to regard 45 mL/min/per 1.73 m^2^ as the threshold value of G3a/G3b staging and whether dividing CKD stage 3 into G3a/G3b plays a useful role in assessing the prognosis of patients with IgA nephropathy (IgAN) remain unknown. Three hundred and ninety patients from the First Affiliated Hospital of Zhengzhou University and Peking University First Hospital diagnosed with IgAN in CKD stage 3 were enrolled and successfully followed up. Cox proportional hazards model was used to analyze hazard ratios of reaching the composite endpoints (doubling of serum creatinine, end-stage renal disease: estimated glomerular filtration rate (eGFR) <15 ml/min/per 1.73 m^2^ or renal replacement therapy, or death) for patients with different eGFR and risk factors affecting composite endpoints. The Kaplan–Meier method was used to calculate the cumulative renal survival rate of patients. When eGFR was lower than 45 ml/min/per 1.73 m^2^, the hazard ratio increased sharply for patients in CKD stage 3 who reached the composite endpoints. Renal injury and prognosis were significantly different between patients in the G3a and G3b groups. Stage G3b was a major risk factor affecting prognosis. A threshold value of 45 ml/min/per 1.73 m^2^ appears appropriate to assess the prognosis of IgAN patients with CKD stage 3. Dividing IgAN patients with CKD stage 3 into G3a and G3b is very useful to help understand disease conditions and for predicting the risk for disease progression.

## Introduction

IgA nephropathy (IgAN) is one of the most common types of primary glomerulonephritis worldwide, and especially in China. The annual incidence is at least 2.5/100 000 adults [1]. IgAN is the major cause of end-stage renal disease (ESRD) in many countries, and approximately 20–50% of IgAN patients progress to ESRD within 20 years [2]. In China, IgAN patients account for 45% of patients with primary glomerulonephritis [3], with a particular number of young adults being affected, which impacts heavily on families and society.

Previous studies have found that in CKD patients a lower estimated glomerular filtration rate (eGFR) is associated with an increased risk for all-cause mortality, cardiovascular events, hospitalization, and infection. The increased risks were evident at an eGFR of less than 60 ml/min/per 1.73 m^2^ and substantially increased with an eGFR of less than 45 ml/min/per 1.73 m^2^ [4,5]. As the data suggest different outcomes and risk profiles, CKD stage 3 was divided into categories G3a and G3b in the 2013 Kidney Disease Improving Global Outcomes (KDIGO) guidelines [6]. However, CKD patients with different kidney diseases were involved in the above studies. For IgAN patients with CKD stage 3, whether it is appropriate to regard 45 ml/min/per 1.73 m^2^ as the threshold value of G3a/G3b staging, whether dividing CKD stage 3 into G3a/G3b plays an important role in assessing the risk for all-cause mortality, doubling of serum creatinine, or reaching ESRD in IgAN patients remains unclear. To investigate these questions, the current study first analyzed the hazard ratios (HR) of reaching defined composite endpoints among IgAN patients with different eGFR, then compared the clinicopathological data and the prognosis of IgAN patients in G3a and G3b. Finally we assessed whether this new classification was an important risk factor influencing the prognosis of IgAN patients in CKD stage 3.

## Materials and methods

### Patient recruitment

Four hundred and twenty-six patients with biopsy-proven IgAN in CKD stage 3 (30 ml/min/per 1.73 m^2^ ≤eGFR <60 ml/min per 1.73 m^2^) were recruited. One hundred and fifty-three patients were from Peking University First Hospital in a prospective cohort study between January 2003 and June 2013. Two hundred and thirty-seven patients were from The First Affiliated Hospital of Zhengzhou University in a retrospective study between January 2001 and June 2015. Patients with secondary IgAN were excluded, such as systemic lupus erythematosus, rheumatic disease, Henoch-Schönlein purpura, hepatitis B virus-associated glomerulonephritis, and liver cirrhosis.

### Ethical statement

The study was approved by ethics committee of the First Affiliated Hospital of Zhengzhou University and Peking University First Hospital. All participants provided written informed consent prior to study inclusion. The study was conducted in accordance with the principles contained in the Declaration of Helsinki.

### Grouping

eGFR was calculated according to the CKD-EPI equation [7]. Patients were divided into two groups according to 2013 KDIGO guideline criteria [6], these being: G3a: 45 ml/min/per 1.73 m^2^ ≤eGFR <60 ml/min/per 1.73 m^2^; and G3b: 30 ml/min/per 1.73 m^2^ ≤eGFR <45 ml/min/per 1.73 m^2^.

To explore HR of reaching endpoints in patients with different eGFR, patients were classified into six subgroups according to eGFR: 55 to <60 ml/min/per 1.73 m^2^ (group 1); 50 to <55 ml/min/per 1.73 m^2^ (group 2); 45 to <50 ml/min/per 1.73 m^2^ (group 3); 40 to <45 ml/min/per 1.73 m^2^ (group 4); 35 to <40 ml/min/per 1.73 m^2^ (group 5); and 30 to <35 ml/min/per 1.73 m^2^ (group 6). Hypertension was defined as systolic pressure ≥140 mmHg and/or diastolic pressure ≥90 mmHg. Nephrotic range proteinuria was defined as ≥3.5 g/24 h. Hemoglobin <120 g/L was determined as anemia for males, and <110 g/L for females. Hyperphosphatemia was defined as >1.45 mmol/L based on 2009 KDIGO guidelines [8]. Treatment groups were divided into two subgroups: treatment without angiotensin-converting-enzyme inhibitors (ACEI)/angiotensin II receptor blockers (ARB) or glucocorticoids/immunosuppressants; and treatment with ACEI/ARB or glucocorticoids/immunosuppressants.

### Follow-up

All patients were followed up according to composite endpoints. Composite endpoints included the doubling of serum creatinine, reaching ESRD (eGFR <15 ml/min per 1.73 m^2^ or renal replacement therapy), or all-cause mortality. Patients with complete medical records were entered into follow-up.

### Renal histopathological grade

Renal sections were evaluated using light microscopy by two experienced pathologists blind to clinical data. The index of mesangial hypercellularity was graded as: 1; mild proliferation (occupying <25% of glomerular area), 2; moderate proliferation (occupying 25–50% of glomerular area), 3; severe proliferation (occupying >50% of glomerular area). Indexes of tubular atrophy, interstitial fibrosis, and cell infiltration were graded as: 0; none, 1; occupying <25% of cortical area of biopsy specimen, 2; involving 25–50% of cortical area, 3; occupying >50% of cortical area [9]. Global and segmental sclerosis, proliferative glomerulosclerosis (glomerular capillary occlusion and glomerular waste caused by mesangial matrix hyperplasia), ischemic glomerulosclerosis (glomerular capillary collapse caused by glomerular ischemia but there is no mesangial matrix hyperplasia), segmental glomerulosclerosis or adhesion, total crescents, cellular crescents, fibrocellular crescents, and fibrous crescents were calculated as the percentage of the total number of glomeruli.

### Statistical analyses

Statistical analyses were performed using SPSS17.0 Statistical Software. Quantitative data of variables with a normal distribution were shown as mean ± standard deviation (SD). Data with a skewed distribution were shown as median and interquartile range. For data with a normal distribution, the Student’s *t*-test was used, otherwise nonparametric tests were used. The chi-square test was used for categorical variables. We derived cumulative kidney survival curves using the Kaplan–Meier method, and analyzed differences between curves using the log-rank test. Univariate and multivariate analysis was performed using the Cox proportional hazards model to identify independent predictor factors for survival of IgAN patients in CKD stage 3. Analysis of Variance (ANOVA) was used in comparing the differences among the six subgroups with different eGFR. *P* value <0.05 (two sided) was considered statistically significant.

## Results

### Clinical and pathological data of IgAN patients in CKD stage 3 with different eGFR

To explore HR of reaching endpoints in patients with different eGFR, patients were classified into six subgroups according to eGFR. Clinical and pathological characteristics of the six subgroups were compared. It was found that hemoglobin, serum uric acid, serum creatinine, serum urea nitrogen, eGFR, serum phosphorus, age, proportions of total crescents, the grade of tubular lesions and grade of interstitial lesions were significantly different among the six subgroups (*P*<0.05). (Tables 1 and 2).

**Table 1:** Clinical characteristics of IgAN patients with CKD stage 3 with different eGFR (mL/min/per 1.73m^2^).

Characteristics	55 to < 60 (n = 70)	50 to <55 (n = 77)	45 to < 50 (n = 63)	40 to < 45 (n = 67)	35 to < 40 (n = 58)	30 to < 35 (n = 55)	*P* value
Sex(male/femal)	46/24	57/20	41/22	47/20	41/17	32/23	0.489
Age (yr)	36.94±11.67	41.70±11.79	39.52±13.05	40.66±12.85	44.93±13.50	36.73±12.32	0.003*
SBP (mmHg)	129.80±18.78	135.61±17.41	135.57±16.47	132.94±17.29	136.21±16.34	137.38±17.94	0.146
DBP (mmHg)	83.39±13.89	87.52±11.86	83.40±11.55	85.67±14.25	86.76±11.99	88.69±10.48	0.086
Hemoglobin (g/L)	129.85± 19.23	134.04 ± 22.27	123.63±21.71	128.47±22.39	113.96±19.85	120.76±27.02	< 0.001*
Serum uric acid (umol/L)	393.43± 85.74	434.00± 103.68	407.13± 112.47	456.97± 133.25	455.12± 118.36	459.53± 119.01	0.001*
Scr (umol/L)	128.19± 27.98	137.77± 20.76	146.65± 25.34	164.10± 31.85	179.19± 32.31	208.02± 46.29	< 0.001*
Serum urea nitrogen (mmol/L)	7.74±2.81	8.04± 3.18	8.72± 3.40	10.51± 3.75	10.40± 3.33	12.21± 5.00	< 0.001*
eGFR(mL/min/per 1.73m^2^)	57.69±1.64	52.64±1.35	47.27±1.41	42.48±1.42	37.67±1.38	32.34±1.39	< 0.001*
Serum albumin (g/L)	35.43±6.68	37.81±6.24	41.72±52.00	34.80±8.35	36.16±6.86	35.49±6.44	0.49
Serum cholesterol (mmol/L)	5.43±1.89	4.84±1.25	5.12±1.82	4.90±1.34	5.31±2.15	4.84±1.35	0.179
Serum triglyceride(mmol/L)	1.94±1.65	1.99±1.52	1.81±1.68	2.62±2.17	1.99±1.20	1.99±1.10	0.076
Serum calcium (mmol/L)	2.23 ± 0.13	2.27 ± 0.14	2.19±0.17	2.20±0.16	2.22±0.21	2.24±0.17	0.078
Serum phosphorus (mmol/L)	1.24 ± 0.25	1.22 ± 0.23	1.27±0.22	1.33±0.38	1.28±0.25	1.48±0.27	< 0.001*
Proteinuria of 24-hour (g)	1.82(3.51,6.16)	1.60(2.60,5.34)	2.05(4.07,7.13)	2.08(3.52,5.26)	2.50(4.93,5.91)	1.96(4.29,6.26)	0.677

*P<0.05.

Abbreviations: IgAN, IgA nephropathy; CKD, chronic kidney disease; eGFR, estimated glomerular filtration rate; SBP, systolic blood pressure; DBP, diastolic blood pressure; Scr, serum creatinine.

**Table 2:** Pathological characteristics of IgAN patients with CKD stage 3 with different eGFR (mL/min/per 1.73m^2^).

Characteristics	55 to < 60 (n = 70)	50 to <55 (n = 77)	45 to < 50 (n = 63)	40 to < 45 (n = 67)	35 to < 40 (n = 58)	30 to < 35 (n = 55)	*P* value
Total crescents (%)	6.91(0.00,18.83)	5.00(0.00,18.18)	10.71(0.00,23.05)	0.00(0.00,12.95)	9.38(0.00,24.52)	12.50(0.00,26.09)	0.007*
Cellular crescents (%)	0.00(0.00,3.72)	0.00(0.00,0.00)	0.00(0.00,1.73)	0.00(0.00,0.00)	0.00(0.00,4.73)	0.00(0.00,4.88)	0.214
Fibrocellular crescents (%)	0.00(0.00,4.76)	0.00(0.00,5.56)	0.00(0.00,11.11)	0.00(0.00,5.91)	0.00(0.00,12.32)	0.00(0.00,13.04)	0.066
Fibrous crescents (%)	0.00(0.00,7.02)	0.00(0.00,4.35)	0.00(0.00,5.88)	0.00(0.00,0.00)	0.00(0.00,4.73)	0.00(0.00,10.00)	0.117
Segmental glomerulosclerosis or adhesion (%)	5.06(0.00,13.33)	0.00(0.00,9.68)	7.14(0.00,14.29)	7.69(0.00,17.40)	5.56(0.00,14.29)	5.26(0.00,13.33)	0.538
Ischemic glomerulosclerosis (%)	5.36(0.00,25.00)	12.96(0.00,33.33)	5.26(0.00,30.39)	15.38(0.00,35.07)	14.84(0.00,37.94)	23.81(0.00,43.48)	0.199
Proliferative glomerulosclerosis (%)	0.00(0.00,9.02)	0.00(0.00,11.11)	0.00(0.00,8.12)	0.00(0.00,12.60)	0.00(0.00,12.32)	0.00(0.00,18.52)	0.158
The grade of tubular lesions (0/1/2/3, %)	(4.29%, 44.29%, 37.14%, 14.29%)	(2.69%, 51.95%, 32.47%, 12.99%)	(3.17%, 41.27%, 38.10%, 17.46%)	(0.00%, 34.33%, 34.33%, 31.34%)	(0.00%, 25.86%, 51.72%, 22.41%)	(1.82%, 23.64%, 34.55%, 40.00%)	< 0.001*
The grade of mesangial proliferative (1/2/3, %)	(87.14%, 8.57%, 4.29%)	(89.61%, 9.09%, 1.30%)	(90.48%, 6.35%, 3.17%)	(83.58%, 14.93%, 1.49%)	(84.48%, 10.34%, 5.17%)	(83.64%, 16.36%, 0.00%)	0.425
The grade of interstitial lesions (0/1/2/3, %)	(4.29%, 34.29%, 34.29%, 27.14%)	(2.60%, 40.26%, 41.56%, 15.58%)	(3.17%, 20.63%, 33.33%, 42.86%)	(1.49%, 13.43%, 38.81%, 46.27%)	(1.72%, 18.97%, 46.55%, 32.76%)	(0.00%, 9.09%, 38.18%, 52.73%)	< 0.001*

*P<0.05.

Abbreviations: IgAN, IgA nephropathy; CKD, chronic kidney disease; eGFR, estimated glomerular filtration rate.

### HR of reaching composite endpoints for IgAN patients in CKD stage 3 with different eGFR

**Fig 1:**
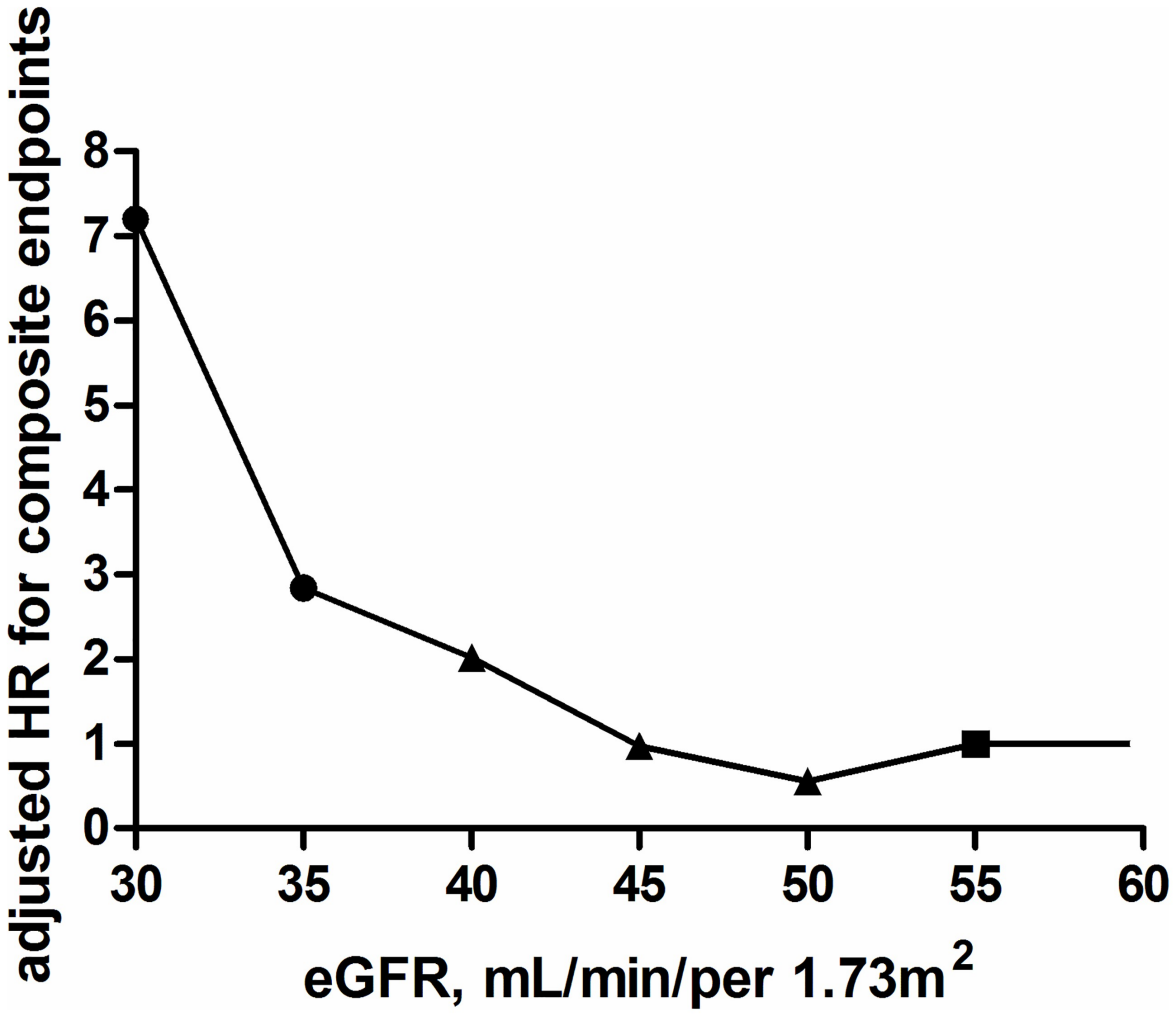
Hazard ratios of composite endpoints in IgAN patients with CKD stage 3 with different eGFR. The reference was eGFR 55–60 mL/min/per 1.73 m^2^. The number of patients in each group was as follows: 55 to < 60: n = 70; 50 to <55: n = 77; 45 to < 50: n = 63; 40 to < 45: n = 67; 35 to < 40: n = 58; 30 to < 35: n = 55. HRs adjusted for age, sex, systolic blood pressure, diastolic blood pressure and proteinuria. Dots represent statistical significance, triangles represent non-significance, and squares represents the reference. Composite endpoints: doubling of serum creatinine, ESRD, or all-cause mortality. Abbreviations: IgAN, IgA nephropathy; CKD, chronic kidney disease; eGFR, estimated glomerular filtration rate; HR, hazard ratios.

Group 1 (eGFR 55 to <60 ml/min/per 1.73 m^2^) was used as the reference group (Table 3). HR for the six subgroups of IgAN patients in CKD stage 3 were: 0.557 (95% confidence interval (CI): 0.225–1.377), 0.974 (95% CI: 0.380–2.494), 2.019 (95% CI: 0.950–4.292), 2.846 (95% CI: 1.330–6.094), 7.201 (95% CI: 3.570–14.523), When the eGFR was lower than 45 ml/min/per 1.73 m^2^, the HR increased sharply for IgAN patients in CKD stage 3 reaching the composite endpoints. This indicated that 45 ml/min/per 1.73 m^2^ may be the threshold value to assess the prognosis of IgAN patients with CKD stage 3. The risk for reaching the composite endpoints dramatically increased when eGFR decreased to 35 ml/min/per 1.73 m^2^, (Table 3 and Fig 1).

**Table 3:** Hazard ratios and 95% confidence intervals for the composite endpoints in IgAN patients with CKD stage 3 with different eGFR.

eGFR (mL/min/per1.73m^2^)	number	HR(95% CI)	*P* value
55 to < 60 †	70	1.000	—
50 to <55	77	0.557 (0.225–1.377)	0.205
45 to < 50	63	0.974 (0.380–2.494)	0.955
40 to < 45	67	2.019 (0.950–4.292)	0.068
35 to < 40	58	2.846 (1.330–6.094)	0.007*
30 to < 35	55	7.201 (3.570–14.523)	< 0.001*
*P* for trend	—	—	< 0.001*

HRs and 95% CIs adjusted for age, sex, systolic blood pressure, diastolic blood pressure and proteinuria. Composite endpoints: doubling of serum creatinine, end-stage renal disease, or all-cause mortality.

*P<0.05.

^†^ This group served as the reference.

Abbreviations: IgAN, IgA nephropathy; CKD, chronic kidney disease; eGFR, estimated glomerular filtration rate; HR, hazard ratios.

### Clinical data of IgAN patients with CKD stage G3a/G3b

Of the 426 participants, 390 completed the follow-up. The age at biopsy ranged from 17 to 80 years old. Mean follow-up time was 41.82 ± 25.90 months. Compared with IgAN patients in CKD stage G3a, creatinine, urea nitrogen, blood uric acid, serum phosphate, and triglycerides were significantly elevated in IgAN patients in CKD stage G3b (*P*<0.05). Hemoglobin and eGFR were significantly decreased in IgAN patients in CKD stage G3b compared with IgAN patients in CKD stage G3a (*P*<0.05) (Table 4).

**Table 4:** Clinical characteristics of IgAN patients with CKD stage G3a and G3b.

Characteristics	stage G3a (n = 209)	stage G3b (n = 181)	*P* value
Age(yr)	39.39 ± 12.23	40.91 ± 13.24	0.242
Sex (male/femal)	143/66	121/60	0.741
Systolic blood pressure (mmHg)	134.00 (120.00, 145.00)	134.00 (120.5, 145.00)	0.350
Diastolic blood pressure (mmHg)	83.00 (78.00, 90.00)	86.00 (80.00, 96.00)	0.065
Hemoglobin (g/L)	129.62 ± 21.49	121.51 ± 23.85	< 0.001*
Serum uric acid (umol/L)	412.80 ± 101.92	456.35 ± 123.70	< 0.001*
Serum creatinine (umol/L)	137.00 (119.70, 150.00)	178.00 (154.00, 204.00)	< 0.001*
Serum urea nitrogen (mmol/L)	7.50 (6.19, 9.09)	10.00 (8.20, 12.70)	< 0.001*
eGFR(mL/min/per 1.73m^2^)	52.68 ± 4.39	37.95 ± 4.65	< 0.001*
Serum albumin (g/L)	37.80 (32.30, 40.65)	37.10 (32.20, 40.50)	0.239
Serum cholesterol (mmol/L)	4.73 (4.11, 5.79)	4.75 (3.88,5.72)	0.747
Serum triglyceride(mmol/L)	1.51 (1.09, 2.11)	1.67 (1.19, 2.90)	0.013*
Serum calcium (mmol/L)	2.23 ± 0.15	2.22 ± 0.18	0.508
Serum phosphorus (mmol/L)	1.24 ± 0.23	1.36 ± 0.32	< 0.001*
Proteinuria of 24-hour (g)	1.78 (1.03, 3.10)	2.10 (1.15, 4.23)	0.082

*P<0.05.

Abbreviations: IgAN, IgA nephropathy; CKD, chronic kidney disease; eGFR, estimated glomerular filtration rate.

### Histopathological parameters of IgAN patients with CKD stage G3a/G3b

Proportions of renal tubulointerstitial injury, mesangial proliferation, and ischemic glomerulosclerosis were greater in patients with CKD stage G3b compared with CKD stage G3a (*P*<0.05) (Table 5). This suggested that kidney injury was more severe in patients with CKD stage G3b compared with CKD stage G3a.

**Table 5:** Pathological characteristics of IgAN patients with CKD stage G3a and G3b.

Characteristics	stage G3a (n = 209)	stage G3b (n = 181)	*P* value
Total crescents (%)	7.69 (0.00, 18.75)	5.41 (0.00, 20.69)	0.945
Cellular crescents (%)	0.00 (0.00, 0.00)	0.00 (0.00, 3.45)	0.317
Fibrocellular crescents (%)	0.00 (0.00, 7.69)	0.00 (0.00, 8.70)	0.626
Fibrous crescents (%)	0.00 (0.00, 5.56)	0.00 (0.00, 4.35)	0.752
Segmental glomerulosclerosis or adhesion (%)	5.00 (0.00, 12.50)	5.88 (0.00, 14.29)	0.382
Ischemic glomerulosclerosis (%)	9.52 (0.00, 30.77)	17.39 (0.00, 40.00)	0.012*
Proliferative glomerulosclerosis (%)	0.00 (0.00, 9.09)	0.00 (0.00, 12.90)	0.133
The grade of tubular lesions (0/1/2/3, %)	(3.33%, 46.19%, 35.71%, 14.76%)	(0.56%, 28.33%, 40.00%, 31.11%)	< 0.001*
The grade of mesangial proliferative (1/2/3,%)	(89.05%, 8.10%, 2.86%)	(83.89%, 13.89%, 2.22%)	0.044*
The grade of interstitial lesions (0/1/2/3, %)	(3.33%, 32.38%, 36.67%, 27.62%)	(1.11%, 13.89%, 41.11%, 43.89%)	< 0.001*

*P<0.05.

Abbreviations: IgAN, IgA nephropathy; CKD, chronic kidney disease.

### Cumulative renal survival rate of IgAN patients with CKD stage 3 reaching the composite endpoints

**Fig 2:**
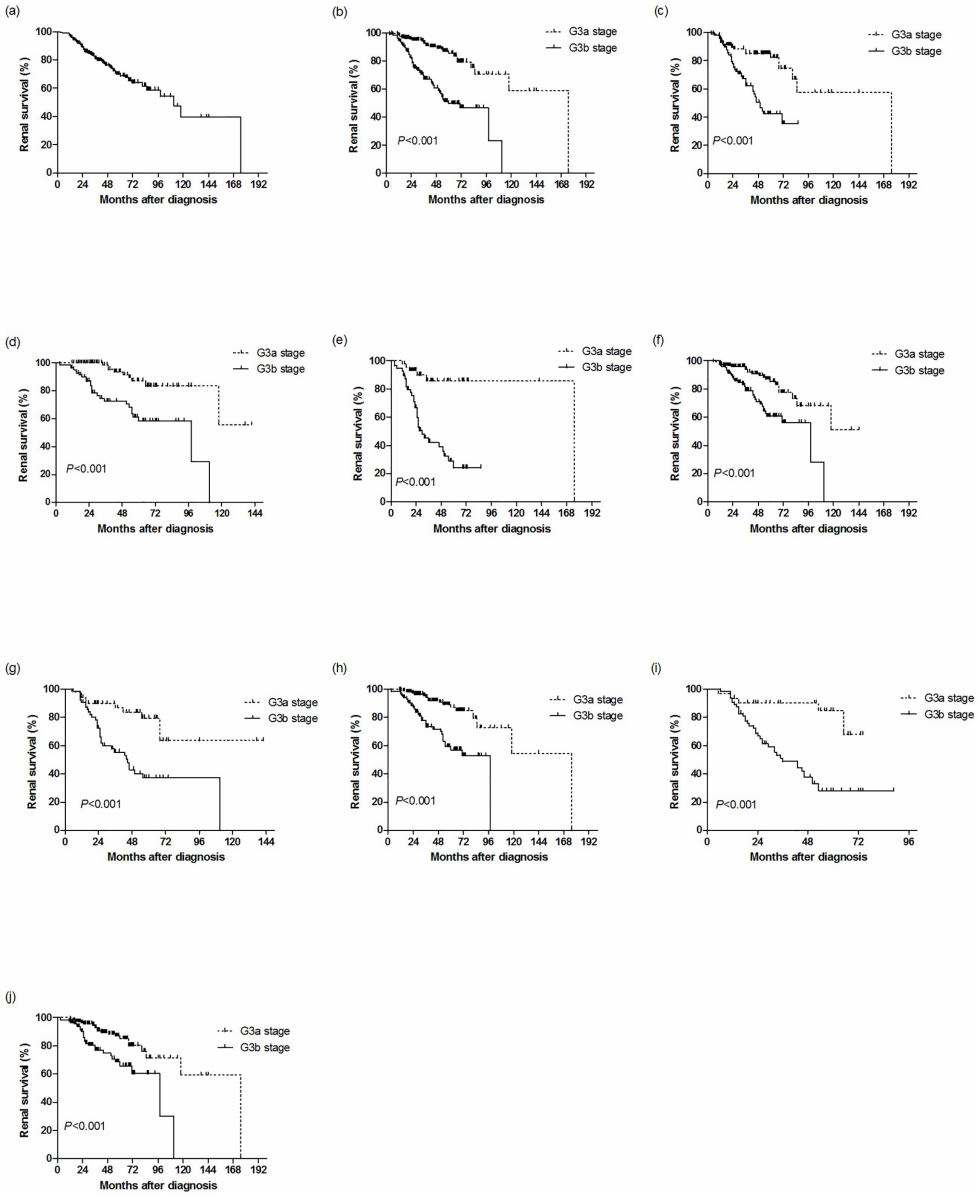
Survival curves of IgAN patients with CKD stage 3 and patients of different subgroups. (a) Patients with CKD stage 3. (b) Patients with CKD stage G3a (dotted line) or G3b (solid line) (log-rank *P*<0.001). Patients with G3a (dotted line) or G3b stage (solid line) based on (c) hypertension (G3a: n = 92, G3b: n = 98) (log-rank P<0.001), (d) non-hypertension (G3a: n = 117, G3b: n = 83) (log-rank P<0.001), (e) proteinuria ≥3.5 g/d (G3a: n = 48, G3b: n = 56) (log-rank P<0.001), (f) proteinuria< 3.5 g/d (G3a: n = 161, G3b: n = 125) (log-rank P<0.001), (g) anemia (G3a: n = 51, G3b: n = 64) (log-rank P<0.001), (h) non-anemia (G3a: n = 158, G3b: n = 117) (log-rank P<0.001), (i) serum phosphorus ≥1.45 mmol/L (G3a: n = 33, G3b: n = 67) (log-rank P<0.001), (j) serum phosphorus <1.45 mmol/L (G3a: n = 176, G3b: n = 114) (log-rank *P*<0.001). Abbreviations: IgAN, IgA nephropathy; CKD, chronic kidney disease.

There were 94 (24.10%) of the 390 IgAN patients with CKD stage 3 that reached the endpoints (Fig 2a). The cumulative renal survival rates of IgAN patients with CKD stage 3 who reached the composite endpoints were 96.41% for l year, 84.36% for 3 years, and 78.46% for 5 years. Of the 209 IgAN patients with CKD stage G3a, 27 patients (12.92%) reached the endpoints. Of the 181 IgAN patients with CKD stage G3b, 67 patients (37.02%) reached the endpoints. The cumulative renal survival rate of IgAN patients with CKD stage G3b was significantly lower than that of patients with CKD stage G3a (*P*<0.01) (Fig 2b). Compared with the G3a group, the cumulative renal survival rates were significantly lower in the G3b group based on hypertension (*P*<0.001) and non-hypertension (*P*<0.001), nephrotic range proteinuria (*P*<0.001) and non-nephrotic range proteinuria (*P*<0.001), anemia (*P*<0.001) and non-anemia (*P*<0.001), hyperphosphatemia (*P*<0.001) and non-hyperphosphatemia (*P*<0.001) (Fig 2c–2j).

### Risk factors affecting renal survival rate of IgAN patients with CKD stage 3 reaching the composite endpoints

To lower the effects of treatment on the prognosis of IgAN patients with CKD stage 3, different treatment subgroups were analyzed. Using the univariate Cox proportional hazards model, we found that stage G3b, hypertension, anemia, nephrotic range proteinuria, hyperphosphatemia, proportion of crescents, proportion of cellular crescents, segmental glomerulosclerosis or adhesion, proportion of proliferative glomerulosclerosis, moderate/severe tubular lesions (grade of tubular atrophy ≥2), moderate/severe interstitial lesions (grade of interstitial fibrosis and interstitial cell infiltration ≥2), and treatment with ACEI/ARB or glucocorticoids/immunosuppressants were all risk factors of IgAN prognosis. These factors as well as gender and age were used in the multivariate Cox proportional hazards model for further analysis. From analysis, the independent risk factors for IgAN patients reaching the endpoints were (in descending order): moderate/severe interstitial lesion, stage G3b, anemia, nephrotic range proteinuria, hypertension, hyperphosphatemia, and proportion of cellular crescents (Table 6).

**Table 6:** Composite endpoints of univariate/multivariate Cox proportional hazards model analysis of IgAN patients with CKD stage 3.

	Univariate analysis		Multivariate analysis	
Variables	HR (95% CI)	*P* value	HR (95% CI)	*P* value
Being in stage G3b	3.970 (2.497–6.313)	< 0.001*	2.540 (1.541–4.187)	< 0.001*
Hypertension	1.865 (1.226–2.838)	0.004*	1.839 (1.149–2.942)	0.011*
Proteinuria ≥ 3.5g/24hours	2.466 (1.621–3.751)	< 0.001*	1.844 (1.170–2.905)	0.008*
Anemia	2.162 (1.436–3.255)	< 0.001*	2.062 (1.327–3.205)	0.001*
Serum phosphorus ≥ 1.45mmol/L	3.269 (2.144–4.982)	< 0.001*	1.801 (1.144–2.836)	0.011*
Cellular crescents (%)	1.037 (1.015–1.060)	0.001*	1.041 (1.017–1.065)	0.001*
Moderate/severe interstitial lesions	4.367 (2.191–8.705)	< 0.001*	3.390 (1.597–7.194)	0.001*
Treatment with ACEI/ARB or glucocorticoids/immunosuppresants	0.491(0.281–0.855)	0.012*		0.480
Moderate/severe tubular lesions	2.112 (1.305–3.417)	0.002*		0.695
Total crescents (%)	1.017 (1.006–1.027)	0.001*		0.985
Segmental glomerulosclerosis or adhesion (%)	1.021 (1.005–1.037)	0.011*		0.311
Proliferative glomerulosclerosis (%)	1.012 (1.000–1.024)	0.047*		0.885

*P<0.05.

Variables tested in the model but not found to be significant included age and sex.

Abbreviations: IgAN, IgA nephropathy; CKD, chronic kidney disease; HR, hazard ratios.

## Discussion

In the 2013 KDIGO guidelines, CKD stage 3 was divided into stage G3a and stage G3b based on different outcomes and risk profiles for the two groups of CKD patients. However, whether this classification can predict the risk for disease progression and evaluate the prognosis for patients with IgAN in CKD stage 3 remain unknown.

Over a median follow-up of 2.84 years of 1 120 295 CKD patients, Alan S Go *et al*. found that mortality dramatically increased in CKD stage 3 when eGFR dropped below 45 ml/min/per 1.73 m^2^ [4]. Levey AS *et al*. found that the relative risk for all outcomes was significantly higher when eGFR was below 45 ml/min/per 1.73 m^2^ in CKD stage 3 patients with the lowest value of albuminuria [10]. However, these studies were based on CKD patients with different kidney diseases. Whether dividing CKD stage 3 into G3a/G3b plays an important role in assessing the prognosis of IgAN patients is seldom studied. The current study is the first to focus only on IgAN patients, and we found that 45 ml/min/per 1.73 m^2^ was the turning point towards a poorer prognosis in IgAN patients with CKD stage 3.

When we compared clinical and pathological data, we found that kidney lesions were more severe in IgAN patients with CKD stage G3b compared with CKD stage G3a, and the cumulative renal survival rate of IgAN patients with CKD stage G3b was lower than that of CKD stage G3a patients who reached the endpoints. Furthermore, the cumulative renal survival rates were lower in the G3b group compared with the G3a group in patients with hypertension or non-hypertension, nephrotic range proteinuria or non-nephrotic range proteinuria, anemia or non-anemia, hyperphosphatemia or non-hyperphosphatemia respectively, indicating a poorer prognosis for patients in stage G3b. These results suggest that renal injury and prognosis were significantly different between IgAN patients in the CKD G3a and G3b groups.

We looked at the risk factors that independently influenced the prognosis of IgAN patients in CKD stage 3. Using the multivariate Cox proportional hazards model, we found that stage G3b, hypertension, nephrotic range proteinuria, anemia, hyperphosphatemia, proportion of cellular crescents, and moderate or severe interstitial lesions were all independent risk factors for IgAN patients reaching the endpoints.

Reich HN *et al*. suggested that proteinuria >1.0 g/d was a significant prognostic indicator of ESRD [11,12] Accordingly, we initially used a Cox proportional hazards model to analyze the hazard ratio of reaching the composite endpoints according to proteinuria 1.0 g/d, and found that proteinuria >1.0 g/d was not a risk factor for IgAN patients reaching the endpoints. This may be related to the high proportion of IgAN patients with proteinuria ≥1.0 g/d in CKD stage 3. Then we divided IgAN patients into two groups according to nephrotic range proteinuria, and found that nephrotic range proteinuria was an independent risk factor for IgAN patients reaching the endpoints, which suggests that reducing proteinuria to <3.5 g/24 h will help delay progression in IgAN patients with CKD stage 3.

In the current study, a Cox proportional hazards model also indicated that hyperphosphatemia was an independent risk factor for IgAN patients reaching the endpoints. Palmer SC *et al*. reported that hyperphosphatemia was an independent risk factor for cardiovascular and cerebrovascular calcification and all-cause mortality in CKD patients [13–15]. In a meta-analysis that included 12 cohort studies with 25 546 patients with CKD, Jingjing Da *et al*. found that a high serum phosphorus level was an independent risk factor for kidney disease progression and mortality among non-dialysis-dependent patients with CKD [16]. Serum phosphorus control is critical for improving the long-term prognosis of CKD patients including IgAN patients with CKD stage 3.

Prior studies have reported that anemia and hypertension are independent risk factors for CKD progression [17,18]. In line with the current study, we found that hypertension and anemia were independent risk factors for composite endpoints. We also found that the proportion of cellular crescents and moderate or severe interstitial lesions were independent risk factors for IgAN patients reaching the composite endpoints.

When compared with other independent risk factors, the question arises as to whether G3a/G3b staging plays a more important role for assessing the prognosis of IgAN patients with CKD stage 3. A multivariate Cox proportional hazards model indicated that the HR for patients at stage G3b progressing to the composite endpoints was 2.54-times greater than those at stage G3a. Compared with other risk factors, the HR of being at stage G3b is only second to moderate or severe interstitial lesions. This finding suggests that stage G3b is a major risk factor affecting prognosis.

In conclusion, we found that an eGFR of 45 ml/min/per 1.73 m^2^ is the turning point for IgAN patients with CKD stage 3 progressing to the composite endpoints. Clinical data, pathological features, and prognosis were significantly different between the two groups (stage G3a and G3b). Stage G3b at kidney biopsy was a major risk factor affecting the prognosis of IgAN patients with CKD stage 3. Dividing IgAN patients with CKD stage 3 into G3a and G3b is very useful to help understand the disease condition and for predicting the risk for disease progression as well as assessing the prognosis of patients with IgAN in CKD stage 3.
